# The influence of talus size and shape on *in vivo* talocrural hopping kinematics

**DOI:** 10.1098/rsos.231997

**Published:** 2024-10-09

**Authors:** Anja-Verena Behling, Luke Kelly, Lauren Welte, Michael J. Rainbow

**Affiliations:** ^1^ School of Human Movement and Nutrition Science, The University of Queensland, Brisbane, Australia; ^2^ Department of Mechanical and Materials Engineering, Queen’s University, Kingston, Canada; ^3^ Australian Centre for Precision Health & Technology, Griffith University, Gold Coast, Australia; ^4^ Department of Mechanical Engineering, University of Alberta, Edmonton, Canada; ^5^ Department of Biomedical Engineering, University of Alberta, Edmonton, Canada

**Keywords:** foot, mechanism, mobility, morphometrics, variability

## Abstract

Talus morphology (shape and size) plays a pivotal role in talocrural joint function. Despite its importance, the relationship between talus morphology, particularly the talar dome, and dynamic, *in vivo* talocrural function is poorly understood. Understanding these form–function relationships in a healthy cohort is essential for advancing patient-specific treatments aimed at restoring function. Nine participants (five females) hopped on one leg while biplanar videoradiography and ground reaction forces were simultaneously collected. Three-dimensional bone models were created from computed tomography scans. Helical axes of motion were calculated for the talus relative to the tibia (rotation axes), and a cylinder was fitted through the talar dome (morphological axis). Bland–Altman plots and spatial angles were used to examine the level of agreement between the rotation and morphological axes. A shape model of 36 (15 females) participants was established, and a cylinder fit was morphed through the range of ±3 standard deviations. The rotation and morphological axes largely agree regarding their orientation and location during hopping. The morphological axes were consistently oriented more anteriorly during landing than the rotation axes. Some shape components affect talar dome orientation and curvature independent of size. This suggests that besides bone size, the shape of the talar dome might influence the movement pattern during locomotion. Our findings may further inform talocrural joint arthroplasty design.

## 1. Introduction

The large variation in morphology of the human talus and the talar dome [1–3] could explain the unique talocrural movement patterns observed across individuals [4–9]. If trauma or chronic diseases such as foot osteoarthritis disrupt this complex morphology–function relationship, surgical interventions may be required. Such surgeries, including talus replacement, often have poor outcomes [10–12]. One factor contributing to the limited success of these treatments could be the poor understanding of how the talus, particularly the talar dome’s morphology (size and shape), relates to talocrural joint function [13–15].

Size is the largest source of overall shape variation in the human talus [1,16,17]. In other joint complexes, such as the wrist, size explains the distal and proximal location of the axis of rotation [18,19]. Based on observations at the wrist, we might expect to observe that smaller tali would have a more superiorly located rotation axis than larger tali (e.g. closer to the tibia). Understanding the curvature of the talar dome is important because it might affect moment arms, ligament and tendon resting lengths, and articular contact stress [20,21]. However, the curvature of the talar dome may also vary across individuals independent of size. In other words, the shape of the talus may also influence the location of the axis. While various complex geometric shapes accurately match the talar dome [1,2,22,23], a simple cylinder fit approximates its curvature well [22–24]. Thus, an axis based on the cylinder’s morphology axis may serve as a reasonable proxy to describe the changes related to variations in size and shape due to alterations in the curvature of the talar dome.

While the talar dome’s curvature due to size or shape variation may explain the superior/inferior location of the rotation axis, talocrural motion may be more difficult to capture by a single morphology-based cylinder axis. If the talocrural joint behaves as a hinge (not necessarily only positioned in the sagittal plane only but potentially skewed) and the mediolaterally symmetric changes in sagittal curvature are the primary drivers of talocrural motion, we would expect the orientation of the cylinder and rotation axes to be in a close agreement. It is unclear if the cylinder axis captures mediolateral asymmetric variation and curvature changes about the transverse and coronal planes. Understanding how well the orientation of the rotation axis and cylinder axis agree may provide insight into the aspects of talar dome morphology which are important to talocrural function.

This study aims to quantify whether the morphological axis based on a cylinder fit captures the talus’s rotation axis and to determine the morphological determinants (size and shape) of these axes. Here, we use a novel approach that links *in vivo* kinematics from biplanar videoradiography (BVR) to geometric morphometrics. High-resolution BVR offers the opportunity to accurately and non-invasively measure dynamic, weight-bearing and bone-level motion, mimicking real-life scenarios [7–9,20,25]. While walking and running are more typical in daily life, we chose hopping as our kinematic task because it maximizes the assessed range of motion at the talocrural joint. Based on the literature, we expect that there will be good agreement across participants in the morphological and rotation axes. We also predict that the axes’ superior/inferior location will vary as a function of talus size, where a larger talus corresponds to a further inferiorly positioned axis location relative to the tibia. Finally, we determine which shape features, independent of size, affect the location and orientation of the rotation and morphological axes.

## 2. Methodology

### 2.1. Overview and participants

After institutional review board approval from Queen’s University and written informed consent was obtained, nine healthy participants (five females, mean ± s.d., mass 71.3 ± 14.5 kg, height 171.4 ± 10.7 cm) with no history of lower limb injury hopped barefoot on their right leg while matching their hopping frequency to a metronome at 156 b.p.m. BVR (125 Hz, range of 70−80 kV, 100−125 mA) and synchronized ground reaction forces (1125 Hz, AMTI Optima, AMIT, USA) were collected for three hops during each sustained hopping trial.

### 2.2. Data processing: kinematics derived from biplanar videoradiography

A computed tomography scan (120 kV, 60mA, model: Lightspeed 16, *n* = 9, General Electric Medical Systems, USA) was obtained from the right foot while participants lay prone with their feet plantar flexed (average resolution: 0.356 × 0.356 × 0.625 mm). Tibia and talus bones were segmented (Mimics 24.0, Materialise, Belgium), yielding bone surface meshes and partial volumes (an image volume of the isolated bone of interest) [3].

We used an established processing workflow to track foot bones with BVR [26]. Briefly, the high-speed cameras were calibrated using a custom calibration cube, and the images were undistorted [4] using custom software (XMALab, Brown University, USA) [5]. The translation and orientation of the tibia and talus were measured by matching digitally reconstructed radiographs generated from the partial volumes to two calibrated radiographs for each data frame. Tracking was done in Autoscoper (Brown University, USA) [3].

Gait events were defined using a 15 N threshold in the vertical ground reaction force to determine touchdown and take-off. The transition point between the landing and push-off phase was determined at the active peak (maximal vertical ground reaction force after impact peak). Landing described the phase from touchdown to the transition point and push-off from the transition point to take-off.

The coordinate systems for the tibia and talus were shape-based, and their primary axes were determined by a cylinder fit through the distal tibia [8,9] and talar dome surfaces [8], respectively. For the tibia, the superiorly pointing axis was based on the cylinder orientation from a cylinder fit through the tibia shaft, and the anterior–posterior axis was obtained as the cross-product of the superior and primary axes. For the talus, a sphere was fitted through the talar head, another sphere through the talus’s calcaneal facet and the subtalar axis ran through the centroid of both spheres. The cross-product of the primary and subtalar axes determined the superior axis. Lastly, the anterior–posterior axis was recalculated via the cross-product of the superior and primary axes. The origins were in the centroid of each bone. The coordinate system axes were relabelled such that the *x*-, *y*- and *z*-axes approximate dorsiflexion, inversion and adduction, respectively, with reference to the right foot and right-hand rule [7,8].

We calculated finite helical axes for the talocrural joint resolved in the tibia coordinate system between two time points [9] (electronic supplementary material, figure S1). This resulted in two hopping phases: the landing phase (from touchdown to the transition point) and the push-off phase (from the transition point to toe-off). Each hopping phase corresponds to one helical axis. A helical axis is defined by the rotation about and translation along an axis, termed the rotation axis [18,19]. The axis is also located at a point in space that is dictated by the radius of curvature about which the talus is rotating.

### 2.3. Statistical shape model of talus

We created a shape atlas for the talus bone to quantify differences in talus shapes. Data from multiple institutional review board-approved studies at Queen’s University were pooled together, and written informed consent from all participants was granted prior to data, resulting in 36 tali (15 females, mean ± s.d., mass 73.1 ± 13.7 kg, height 171.5 ± 8.2 cm). A reference mesh was selected after visually inspecting all bones for what we qualitatively determined to be average features. All tali were aligned to their inertial axes and followed by an iterative closest point and rigid coherent point drift (CPD) algorithm to ensure optimal alignment to the reference bone. The first non-rigid CPD was performed to register the meshes to the reference bone by selecting corresponding points on the bone meshes relative to the reference mesh while conserving the individual bone shape. A generalized Procrustes approach was performed to scale and further align the meshes in Procrustes space [10]. Following this, a new reference mesh was calculated as the average mesh across all Procrustes meshes (arithmetic mean). Subsequently, a second non-rigid CPD was performed to register all Procrustes meshes to the new reference bone. The corresponding meshes were used as input for a principal component analysis (PCA). The electronic supplementary material can provide an overview of the steps (electronic supplementary material, figure S2). To separate the effects of shape and size on the rotation axes location and orientation, all further shape analyses (such as the cylinder fit) were carried out on the PCA, where size has already been removed. For each principal component (PC) explaining the modes of variation within the PCA, the mean bone and bone deviations across ±3 s.d. were reconstructed and visualized.

We calculated talus size using centroid size, which is the square root of the sum of the squared distances of all points of the bone mesh from their centroid, to understand the importance of bone size on the rotation axes’ location and orientation.

### 2.4. Cylinder fit through talar dome

**Figure 1.**
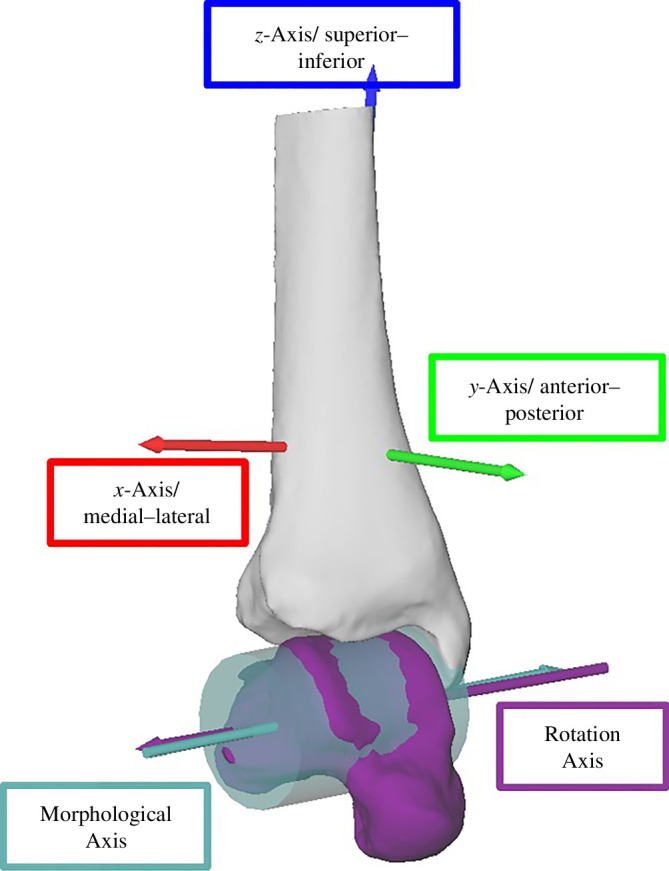
Cylinder fit to talar dome with the morphological axis (teal) and the mean rotation axis for landing (purple) (*n*_Participant_ = 1; *n*_Trials_hopping_ = 3). Tibia coordinate system included (red, green and blue arrow).

A talar dome template region was manually segmented for the new mean bone of the statistical shape model using Geomagic Wrap 2021.2.2. (3D Systems GmbH, Germany), which resulted in a three-dimensional mesh of 180 points. Non-rigid CPD was used to determine the corresponding points on all participants’ talar domes based on the template region and shape morphs. The corresponding talar dome points were then used as the input to fit a point cloud to a cylinder and further optimized using a least square cylinder fit method (version R2023a, Mathworks, USA; figure 1). The orientation and location of the cylinder (morphological axis) in the tibia coordinate system were assessed for each hopping participant (*n* = 9). To calculate the location of the morphological and rotation axis, the tibia coordinate system’s origin was moved to the tibial dome along the superior axis of the coordinate system. This was because the location of the original tibia coordinate system was influenced by how much tibia shaft was available in the CT scan and might, therefore, influence the outcome. The location of the morphological axis in superior–inferior and anterior–posterior directions was calculated relative to the origin of the readjusted tibia coordinate system origin. Therefore, the cylinder’s curvature and talar dome curvature are the inverse of their radii. Here, we reported the radius because it allowed us to compare the rotation axis directly.

### 2.5. Analysis

Bland–Altman analyses determined the agreement between the morphological and rotation axes. We compared the axes’ orientation and reported the spatial angles between the morphological and rotation axes.

To determine the effect of size on talar dome curvature, we performed a linear regression of the cylinder radius with talus size. To determine the effect of talar dome size on rotation axis location, we regressed the vertical location of the rotation axis (the intersection point of the rotation axes with the sagittal plane of the tibia coordinate system) on the centroid size of the respective tali.

To determine the effect of shape on talar dome curvature, first, we applied a parallel analysis to determine the number of PCs significantly different from chance to determine the PCs of interest [11]. From the remaining PCs, each PC explaining more than 10% of the overall variance was visually inspected for changes in the shape of the talar dome, respectively. Then, we regressed the PC loadings of the PCA on the cylinder radius.

The level of significance was set to 0.05.

## 3. Results

### 3.1. Agreement of morphological and rotation axes

**Figure 2.**
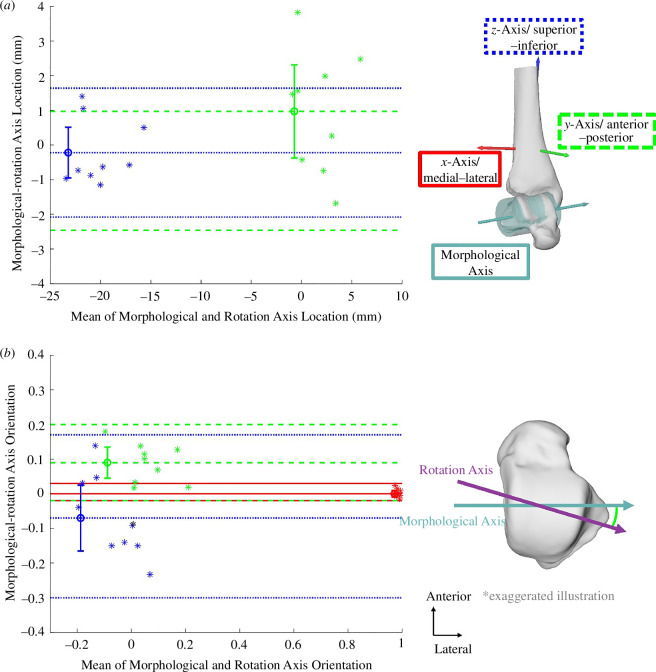
Directions in Bland–Altman plots are colour-coded according to the legend. The level of agreement between the morphological and rotation axis location (*a*) and orientation (*b*) during landing. The line with the error bars indicates the overall mean ± 95% confidence interval, and the other two lines indicate the limits of agreement. Note, there are no medial–lateral values for the location as the locations of the rotation axes were evaluated based on where they penetrate the sagittal plane of the tibia coordinate system. Significant bias in *y*-axis/anterior–posterior direction (green lines in *b*) is illustrated schematically next to panel *b*.

Agreement between the morphological and rotation axes was high for the landing and the push-off phases with mean ± s.d. spatial angles of 9.6° ± 3.5° during landing and 9.0° ± 3.4° during push-off (electronic supplementary material, table S1). Hence, we only report the landing phase here (figure 2); subject-specific figures and results for the push-off phase can be found in the electronic supplementary material, figures S3–S5. The morphological and rotation axes agreed in the anterior–posterior and superior–inferior axis locations as there was no systematic bias, and the limits of agreement were within ±2 mm. The orientation of the morphological and rotation axes agreed best in the medial–lateral direction (indicating rotation in the sagittal plane, which was also their main direction), as there was no systematic bias, and the limits of agreement were narrow (±0.05). The morphological axes systematically were more anterior (bias = 0.1) than the rotation axes during landing (figure 2*b*), and the limits of agreement were wider (±0.1) than in the medial–lateral direction. The orientation in the superior–inferior direction (rotation in the transverse plane) showed no systematic bias but reported the widest limits of agreement (±0.2), indicating the poorest agreement in this direction.

### 3.2. Effect of size and shape on talus dome curvature

When looking at the influence of bone size on talar dome radius, larger tali were associated with larger cylinder radii (*p* = 0.002, *r* ^2^ = 0.61, alpha = 0.05, electronic supplementary material, figure S6). However, larger tali were not associated with lower rotation axes relative to the tibia (electronic supplementary material, figure S7). The first three PCs of the PCA explained 39% of the overall variance within the dataset of scaled tali. Each PC explained more than 10% of the variance. None of the PCs were associated with centroid size (*p* ≥ 0.20, *r* ^2^ ≤ 0.02, alpha = 0.05).

**Figure 3.**
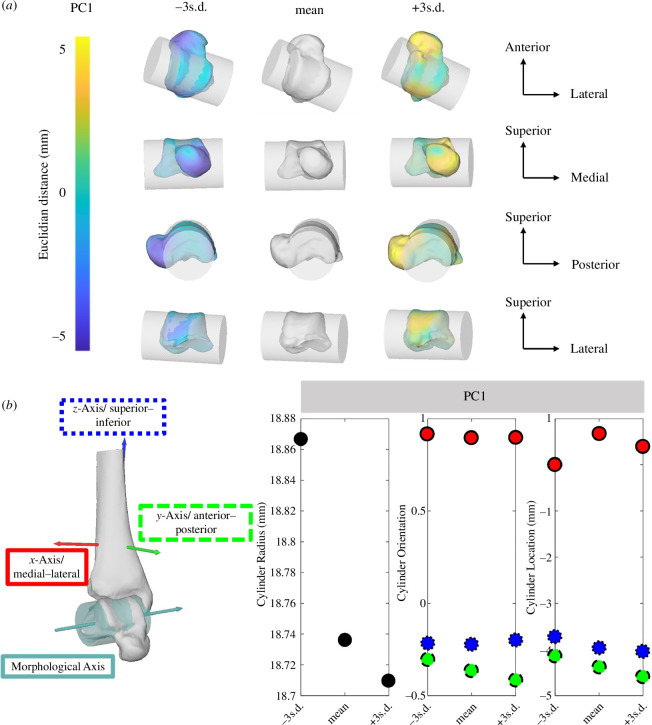
First PC of talus visualized with cylinder fit through talar dome ±3 s.d. from the mean shape. (*a*) Shape changes are colour-coded according to the Euclidean distance in mm to the mean shape. Cylinder metrics for PC1 depicted across ±3 s.d. for changes in cylinder radius (black), orientation and location with the three directions. (*b*) Medial–lateral (red and solid outline), anterior–posterior (green and dashed outline) and superior–inferior (blue and dotted outline; *n*_Participant_ = 36).

PC1 explained 16% of the overall variance in the dataset and described changes in talus posterior process prominence (shortening and lengthening), talus head orientation (twisting of the talus head), and width and length of the posterior and anterior section of the talar dome facet (figure 3*a*). Within this PC across 3 s.d., alterations in talar dome metrics (cylinder radius and location) were within 1 mm (figure 3*b*). The changes in cylinder and morphological axis orientation were below ±0.01 in the medial–lateral and superior–inferior direction and small in the anterior–posterior direction (±0.1).

**Figure 4.**
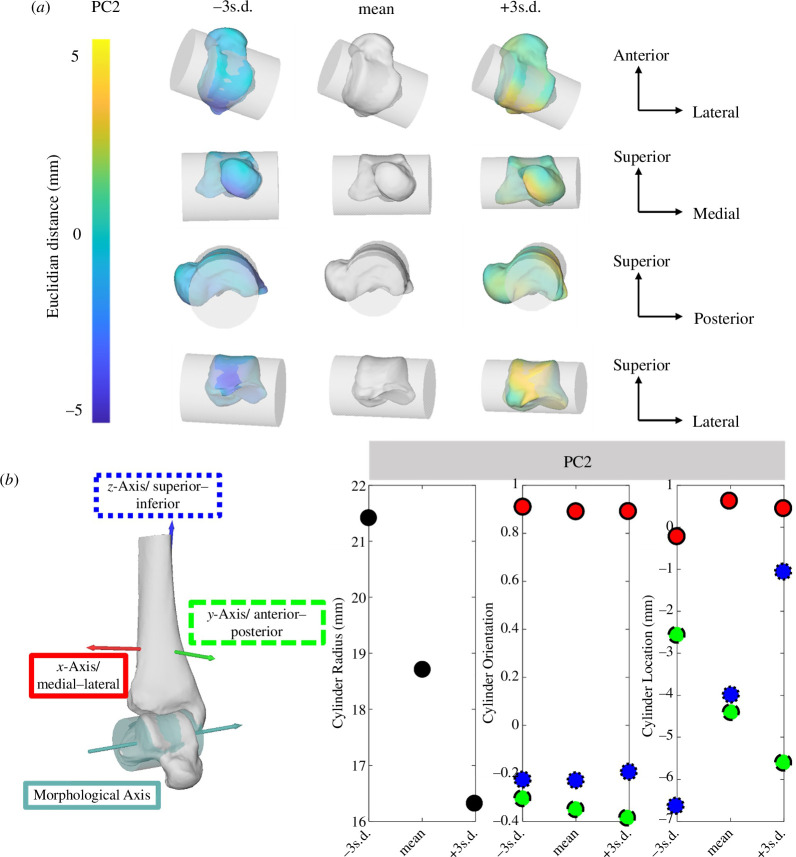
Second PC of talus visualized with cylinder fit through talar dome ±3 s.d. from the mean shape. (*a*) Shape changes are colour-coded according to the Euclidean distance in mm to the mean shape. Cylinder metrics for PC2 depicted across ±3 s.d. for changes in cylinder radius (black), orientation and location with the three directions. (*b*) Medial–lateral (red and solid outline), anterior–posterior (green and dashed outline) and superior–inferior (blue and dotted outline; *n*_Participant_ = 36).

PC2 explained 12% of the overall variance in talus shape. The main morphological variations can be seen in the posterior process prominence that ‘tucks under’ the posterior talus region (altering the curvature of the talar dome) and lateral side of the talar head (shape changes indicated in dark blue and yellow across ±5 mm s.d., figure 4*a*). Consequently, the more curved the talar dome, the smaller the cylinder radius and the more superiorly and posteriorly located the morphological axis (associated here with +3 s.d. changes, figure 4*b*). The changes in talar dome orientation occurred in the superior–inferior direction (internal–external rotation) but were small (±0.1) and even smaller in the medial–lateral direction.

**Figure 5.**
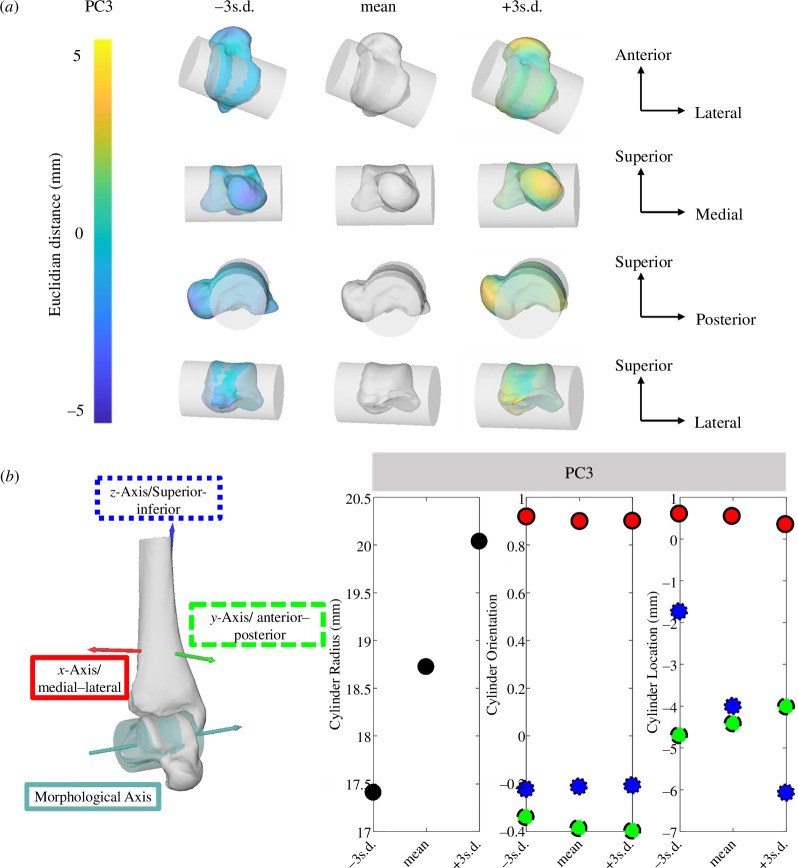
Third PC of talus visualized with cylinder fit through talar dome ±3 s.d. from the mean shape. (*a*) Shape changes are colour-coded according to the Euclidean distance in mm to the mean shape. Cylinder metrics for PC3 depicted across ±3 s.d. for changes in cylinder radius (black), orientation and location with the three directions. (*b*) Medial–lateral (red and solid outline), anterior–posterior (green and dashed outline) and superior–inferior (blue and dotted outline; *n*_Participant_ = 36).

PC3 explained 11% of the overall variance in the talus. The changes were most prominent in the posterior aspect of the talus (posterior talar dome surface) and most anterior aspect of the talus head (bulging of talus head), which appear to elongate or shorten the talus (figure 5*a*). The posterior shape alterations especially affect the size of the cylinder, hence the talar dome curvature, and the morphological axis location in the superior–inferior direction (figure 5*b*). For example, the shorter (missing the posterior process) but flatter talar dome (here associated with +3 s.d., figure 5*a*), the larger the cylinder radius is, and the more inferiorly located the morphological axis (figure 5*b*). The changes in morphological axis orientations were below ±0.1 in all three directions.

Since PC1 of the PCA did not affect the cylinder radius, no regression was performed between PC1 loadings and the cylinder radius. However, regressions for PC2 and PC3 loadings of the PCA and cylinder radius showed significant relationships (electronic supplementary material, figure S8). Cylinder radius decreased with larger PC2 loadings (*p* = 0.0001; *r* ^2^ = 0.09, electronic supplementary material, figure S8A), while the radius increased with larger PC3 loadings (*p* = 0.0001; *r* ^2^ = 0.47, electronic supplementary material, figure S8B).

## 4. Discussion

This study aimed to quantify the agreement of the morphological (based on a cylinder fit) and rotational axes of the ankle joint and to determine the morphological features that influence these axes. Overall, we found that the rotation axis was tightly coupled to the morphological axis; however, the morphological axis consistently overestimated the anterior–posterior orientation of the rotation axis. From the shape atlas of the talus, we found that both size and morphological features have independent influences on the curvature of the talar dome and, therefore, the location of the morphological axis, as determined by the cylinder fit. The most striking morphological features across the first three PCs in the shape model showed large variation in the prominence of the talus’s posterior section and the talar dome’s anterior–posterior length in addition to the twisting of the talar head [5,16–20].

Fitting a cylinder to the talar dome served two purposes; first, it allowed us to test the assumption that the talocrural joint acts as a simple hinge (not necessarily only in sagittal plane orientation). Second, it helped simplify the description of the complex shape features captured in our statistical shape model. While the rotation and morphological axes agree well regarding the axis location, the agreement in orientation was poorer. The morphological axis consistently predicted more talocrural inversion–eversion than was present. This finding indicates that aspects of the talus geometry dictate talus motion that a simple cylinder cannot capture. These findings also confirm previous observations [1,2] that the talocrural joint does not behave as a simple hinge despite most motion occurring in the sagittal plane. While the out-of-sagittal plane motions were small, they contributed to the deviations in the alignment of the morphological and rotation axes. Taking non-sagittal talar dome morphology into account and capturing a greater range of movement, such as cutting manoeuvres, might provide a more comprehensive assessment of the non-sagittal relationships between morphology and kinematics.

Despite the small anterior–posterior offset in orientation, the agreement between the axes in terms of location and orientation was sufficient to meet our second goal of using the cylinder fit to help describe how variation in size and shape of the talus alters the morphological axes. We were surprised by how well the cylinder captured the function of the talocrural joint, considering that other papers deemed a cylindric shape too simple for the talocrural joint [1,2]. The differences in interpretation might be because these studies did not compare their morphological axes to the rotation axes. Instead, they assessed how well their geometrical shapes fit the raw bone shape. We suggest that validating more complex shapes used in these studies [1,2] with *in vivo* kinematics may be a suitable next step further to understanding the shape–function relationships within the ankle joint.

Talar dome curvature is an important feature for implant design because it may alter the location of the morphological and, therefore, rotation axis, affecting muscle moment arms, contact pressure and muscle-tendon and ligament operating ranges. In our herein-introduced shape model, we captured talar dome curvature by the cylinder fit radius and the morphological axis’s vertical location. Since the morphological and rotation axes’ locations agree well, we assume that alterations in the superior–inferior rotation axes’ location of the talocrural joint accompany changes in the talar dome curvature via size or shape changes. Conceptually, a less curved talar dome corresponds to a larger cylinder, hence a lower vertical location of the morphological and rotation axes, resulting in larger moment arms for tendons and muscles attaching to the talus. This would lead to a larger mechanical advantage and larger tendon excursions for the same angular displacement. According to the force–length relationship, larger tendon excursions result in higher muscle velocities, which lead to reduced forces [20,21]. Ligament and tendon operating ranges may also increase, potentially exposing the tissues to increased strain. The opposite behaviour could occur when the talar dome curvature of the implant is too small for the individual (more curved talar dome). We found that overall talus size is strongly linked to talar dome curvature, indicating that larger bones typically have larger talar domes. This makes sense; however, we also found that a substantial proportion of the variation in the superior–inferior location of the morphological axis was explained by shape features independent of size. This is particularly interesting for implant design, as the bone might not scale isometrically across all features. While more research is required to confirm the consequences of mismatching talar dome curvature, these findings support the emerging practices of developing person-specific implants [12].

Our analysis suggests that the morphological features of the talus (PC2, PC3 and talar dome curvature) do not reliably predict the level of agreement between morphological and rotation axes on a subject-specific level. For instance, if we compare two extreme participants in shape (participants 7 and 9 in electronic supplementary material, figure S8), we notice that participant 7 has a less pronounced talar dome and a more average talus shape than participant 9. Surprisingly, participant 7 exhibits good agreement between the morphological and rotation axes (electronic supplementary material, figures S4 and S5), indicating that their talar shape might promote alignment of the axes. However, participant 5 has similar talar dome curvature and PC2/PC3 loadings to participant 7 but does not show the expected high agreement, suggesting that a specific talar shape does not necessarily determine the level of agreement. However, these interpretations remain speculative because we lack the quantitative sample size to compare shape with the kinematic axes quantitatively. In summary, we found rotation and morphological axes to align well despite the large inter-participant variability regarding their morphology and kinematics.

When interpreting our findings, it is important to consider several limitations. First, we computed our rotation axes over a large range of motion for the dorsi- and plantar flexion phases, respectively. While this improves the robustness of the helical axis metric, it neglects the nuanced motion that occurs during each phase. We could have selected smaller time frames to calculate the helical axes, potentially at the expense of increasing noise in the helical axis orientation [16]. Additionally, as our comparison was with a morphological axis that does not change over the gait cycle, measuring instantaneous motion would not affect our conclusions, and our rotation axis captures the gross motion of the talocrural joint. Second, BVR data are difficult to track, and we were limited to a small sample size. Rather than directly comparing the shape (e.g. weights of PCs) to our rotation axes, we used the cylinder axis as a proxy. Future studies with a larger sample size may be able to gain new insights by directly comparing kinematics with specific shape features and potentially other shape proxies (e.g. cone or bi-truncated cones [2,5,10]). Third, because we wanted to explore how talar dome morphology influenced talocrural kinematics, we did not extend our analysis to include the talus facet on the tibia. We made this simplification based on the assumption that the tibia shape would closely mirror the talar dome shape. However, the talocrural articulation might not fully conform, allowing for rolling and gliding motion that cannot be captured with our current approach [27]. New insights may also be gained by accounting for the tibia surface and the subsequent joint congruence [16]. Fourth, we examined hopping as a model of locomotion (instead of walking or running) to ensure a large range of motion in the talocrural joint per gait phase to compute a robust rotation axis and greater data yield per participant (more frames of data collected). While we have previously shown the kinematic behaviour of the ankle joint to be similar in running and hopping [13], it will be crucial to extend future research to walking and running. Lastly, we would like to point out that PC1−3 explained 39% of the variance in talus morphology. However, considering the complexity of talus articular surfaces and features, reducing the dimensionality to three components while containing 39% of the overall variance is remarkable in our opinion. Despite these limitations, our approach allowed us to determine how well the morphological axis captured the rotation axis and which shape features of the talar dome altered the morphological axes.

### 4.1. Future applications

Understanding how the talocrural joint’s size, shape and motion relate could enhance the effectiveness of ankle joint implants individually tailored to patients undergoing ankle joint arthroplasty. The most common talus prostheses differ in overall size but do not account for individual differences in bone shape, particularly variations in a talar dome shape [1]. This might affect post-surgical ankle joint mechanics, prosthesis lifespan and overall quality of life for patients who undergo these procedures [14,15,25]. If an implant aims to restore ‘natural’ talocrural kinematics, personalizing size and shape features might be an important next step.

However, we cannot make any statement as to whether these allometrically scaled tali implants are ‘better’ than isometrically scaled tali, as this would have to be evaluated in a pre- and post-surgery study. In theory, if we wanted to design a talar dome based on information on the healthy motion of a talocrural joint, we would require an estimation of a rotation axis (e.g. of the contralateral joint if unaffected or ideally of the same joint when it was healthy). We know of no other method other than BVR that can calculate rotation axes at the joint level in a minimally invasive yet dynamic, *in vivo*, and weight-bearing way. Therefore, other technology might only roughly estimate how talar dome morphology is related to talocrural kinematics dynamic tasks. Future studies could compare the accuracy of different technologies (which might be more affordable and accessible) to BVR in measuring our herein-introduced method and/or couple it with musculoskeletal modelling approaches. These future avenues could also investigate how more complex geometric fits perform to replicate talocrural kinematics.

## 5. Conclusion

Here, we have shown that a cylinder fit to the talar dome provides a reasonable estimate of talar dome curvature. Combining this geometric model with high-resolution BVR data, we have revealed that the axis projecting through the centre of the cylinder (morphological axis) aligns closely with the location and, to a lesser extent, the orientation of the rotation axes of the ankle joint. Despite only small offsets in the orientation of the morphological and rotation axes, we report systematic offsets in ankle joint inversion and eversion motion. Thus, small changes in talus morphology and the associated influence on axis orientation produce meaningful kinematic alterations that might greatly affect joint contact forces [28].

## Data Availability

The data can be found on a public server from the University of Queensland via the following link [29]. Supplementary material is available online [30].
